# A Flow-Through Multi-Wavelength Sensor with Machine-Learning Calibration for Real-Time Monitoring of Microalgae Cultivation Processes

**DOI:** 10.3390/s26154913

**Published:** 2026-08-04

**Authors:** Richard Bleisch, Mark D. Komiskey, Sushant Poudel, Luis Porras Reyes, Sascha Beutel, Thomas Walther, Stefan Streif, Felix Krujatz

**Affiliations:** 1 Institute of Natural Materials Technology, Technical University Dresden, 01069 Dresden, Germany; richard.bleisch@tu-dresden.de (R.B.); mark_daniel.komiskey@mailbox.tu-dresden.de (M.D.K.); sushant.poudel@mailbox.tu-dresden.de (S.P.); thomas_walther@tu-dresden.de (T.W.); 2 Institute of Technical Chemistry, Leibniz University of Hanover, 30167 Hanover, Germany; porras@iftc.uni-hannover.de (L.P.R.); beutel@iftc.uni-hannover.de (S.B.); 3 Professorship Automatic Control and System Dynamics, Chemnitz University of Technology, 09126 Chemnitz, Germany; stefan.streif@etit.tu-chemnitz.de; 4 Department of Bioresources, Fraunhofer Institute for Molecular Biology and Applied Ecology, 35392 Giessen, Germany; 5 Biotopa gGmbH—Center for Applied Aquaculture & Bioeconomy, 01454 Radeberg, Germany

**Keywords:** soft sensor, Support Vector Regression (SVR), microalgae, real-time monitoring, *Chromochloris zofingiensis*, flow-through multi-wavelength sensor, carotenoids, astaxanthin

## Abstract

Acquiring real-time biological data is essential for effective control of microalgae cultivation processes, yet routine monitoring still depended on laborious offline analyses that relied on time-consuming wet-chemical techniques. This study introduces a flow-through, multi-wavelength visible-light (VIS) sensor for real-time monitoring of biomass and pigment concentrations in microalgae cultivation processes. Based on 209 experimental data points, six machine-learning regression models were developed to estimate dry biomass, chlorophyll α, chlorophyll β, total chlorophyll, total carotenoid, and astaxanthin concentrations. Validation under realistic continuous operation showed that potential remains within improved hydrodynamics and operation automatics to reduced biofilm formation. The independent dataset demonstrated that biomass and astaxanthin predictions were within ±10% of offline reference measurements. The proposed low-cost and versatile multi-wavelength sensor platform, together with machine-learning-based calibration, provides a practical soft-sensor concept for real-time monitoring of microalgal bioprocesses and offers a foundation for future integration of model-based and predictive control strategies.

## 1. Introduction

This project investigates the potential of a multi-wavelength visible light (VIS) sensor as a soft sensor to monitor the growth and pigment accumulation of the promising microalga *Chromochloris zofingiensis* as a model organism. Due to their immense potential as a viable source of various high-value pigments for food, pharmaceutical, and cosmetic purposes, microalgae processes reach more and more pilot and industrial scale production [1].

Microalgae are a diverse group of mostly autotrophic living microorganisms that produce a variety of organic compounds [2]. Some of these are of high value due to their bioactive properties like oils, carotenoids, proteins, and polysaccharides [3,4,5]. Naturally, there is a growing demand for scalable and sustainable methods of producing microalgae that generate these high-value molecules. The green microalgae *Chromochloris zofingiensis* is a promising candidate for astaxanthin production a highly antioxidative component [6,7]. The process of astaxanthin accumulation is nonlinear. Continuous monitoring supports the definition of an optimal time for harvesting the culture at high biomass or product concentrations. *C. zofingiensis* is a nonmotile, haploid, coccoid, freshwater Chlorophyte [5,8]. The cells are spherically shaped and range in size from 2 to 17 µm, depending on the life stage of cell growth [5]. While *C. zofingiensis* can be used to obtain astaxanthin, other carotenoids such as zeaxanthin, canthaxanthin, lutein, and fucoxanthin are also of interest as well as macro components like lipids, proteins, and carbohydrates.

Having accurate information about the current biological parameters like biomass concentration, biochemical compositions or to identify any potential contamination of the culture in real-time is crucial for the efficient operation of large-scale microalgae production [9,10,11]. Such information is vital for optimizing growth conditions, for process monitoring, for ensuring product quality and for maximizing the yield. In the best case, these biological parameters can be measured in real-time (on-line) and process integrated (in-line) in a noninvasive manner. Typically, the determination of biological parameters relies on sampling followed by laborious offline analyses, commonly employing time-consuming wet-chemical techniques [12]. Quickly advancing new approaches like model-based adaptive control are not feasible with this delayed discontinuous data [13,14,15].

**Figure 1:**
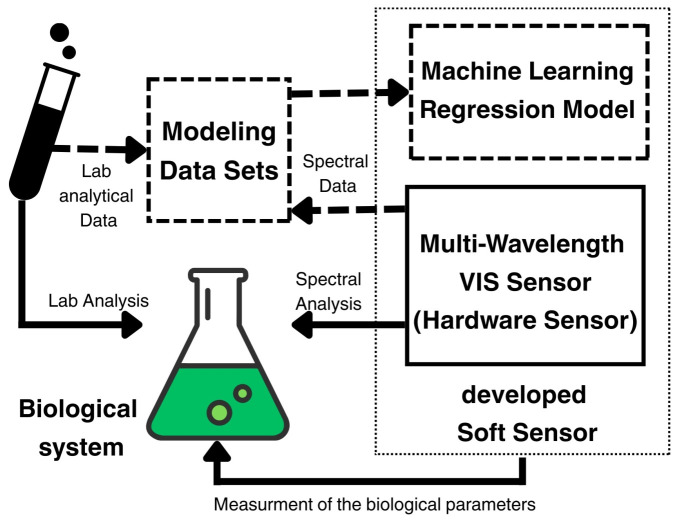
Schematic of the developed soft sensor. The spectral analytical data together with the lab analytical data was used to develop the machine learning regression models and together with the hardware sensor they form the sofware sensor capable to measuring biological parameters of the microalgae system. Solid lines: Measurement of physical chemical or biological parameters of the biological system; dashed lines: Flow of the data.

To address this challenge, indirect on-line measurement techniques can be employed to sample process data in a noninvasive, automatic, continuous way. These indirect measuring sensors are often referred as ’Software Sensors’ or ’Soft Sensors’ [16]. A soft sensor is a combination of a hardware sensor (which measures distinct parameter directly) and an estimation algorithm that deduces the unmeasured parameters of interest from the measured parameters [16]. The schematic of a soft sensor is illustrated in Figure 1 using the example of the multi-wavelength VIS sensor as the hardware component.

Optical methods are among the most widely used techniques for biomass estimation [9,10]. They offer distinct advantages over other physical measurement approaches, particularly when analyzing pigment-containing samples. Additionally, the soft sensor response is immediate without delay and noninvasive. Disadvantages of optical methods are their sensitivity to environment changes like light contamination with variations in the intensity of process or environmental light or the inaccurate alignment of the LED-photodetector setup.

Optical methods include most commonly transmission- and scattering-based approaches. Other optical methods such as color analysis [17,18], near-infrared spectroscopy (NIR) [19,20], in situ microscopy (ISM) [21], or focused beam reflectance measurement (FBRM) [22] can also potentially be used for real-time analysis of microalgae cultures. However, data regarding their suitability is very limited, and the devices are often proprietary and discontinuous, making their integration into continuously operating processes challenging. Available studies for real-time monitoring of microalgae biomass and pigment concentrations are collected in Table 1 and Table 2. Transmission-based approaches have to account for the effect of self-shading in particle based biological systems that always occur at higher biomass dry weight (DW) concentration. Above a certain particle concentration, the measurement system can no longer detect a transmission signal. The scattering-based approaches are less limited by this effect. Scattering includes the physical effects of transmission, reflectance, refraction, dispersion, and diffraction [23], with reflectance (backscatter) here specifically applied to measure high biomass concentrations without dilution [24].

As seen in Table 1 and Table 2, in most cases a single light source with one distinct wavelength and one fixed intensity is used to acquire data. However, to accurately capture variations in pigment composition and biomass across both low and high concentration ranges, measurements at multiple wavelengths combined with varying excitation light intensities are required. Barbosa et al. [34] demonstrated this using a multi-wavelength VIS sensor with adjustable excitation light intensity. Results by Havlik et al. [21] and Borah et al. [17] further indicated the high feasibility of the usage of multiple light excitation sources with different wavelengths. Especially for the measurement of more distinct biological parameters like pigment concentration or macrocomposition, a multi-wavelength VIS sensor appears to be the most suitable choice [17,35].

Most VIS sensors continue to rely on empirical calibrations to measure the biomass concentration. The potential of a data-driven neural-network architecture to predict the biomass concentrations of *Chlamydomonas reinhardtii* had already been demonstrated [31]. With an increasing number of data collected with each individual measurement, the collected data needs to be processed using holistic strategies [17] that are data-driven. Machine-learning approaches such as neural networks, Support Vector Regression (SVR), or linear regression models offer this potential. So far the application of data-driven sensors is not well studied but is gaining more attention. They offer potential not only for measuring higher biomass concentrations but also for capturing more distinctive culture characteristics, such as pigmentation and biomass composition [19,36,37]. Additionally, potential contaminations in the biological system can be detected early and model-based or predictive control can be applied to the process based on the sensor measurements.

## 2. Materials and Methods

### 2.1. Microalgae Strain and Cultivation Conditions

The *Chromochloris zofingiensis* strain was obtained from the Culture Collection of Algae at Göttingen University (SAG 211-14, Göttingen, Germany). The strain was maintained with Bristol’s Modified medium in 250 mL shake flasks with a 50 mL working volume, cultivated at 25 °C, 150 rpm, and a 16:8 h light/dark photoperiod [38].

To obtain a representative dataset for training and testing the machine learning regression models (MLRM) of the soft sensor, *C. zofingiensis* was cultivated under systematically varied cultivation conditions. To enhance compatibility with industrial processes and to minimize model overfitting, data was acquired by cultivating the algae across multiple cultivation systems in batches. These included a multi-cultivation platform with 9 × 150 mL cultivators (CellDEG GmbH, Berlin, Germany), a tubular 25 L-PBR (PUEVIT GmbH, Dresden, Germany) and a tubular pilot-scale 200 L-photobioreactor (PBR) (PUEVIT GmbH, Dresden, Germany). This approach provides sufficient variance in both biomass composition and pigment concentrations across the collected data.

The CellDEG multi-cultivation platform offers optimal growth conditions with bubble-free, membrane-based aeration with ${\mathrm{CO}}_{2}$-enriched air (2% *v*/*v*) at room temperature (22±2 °C).

The pilot-scale PBR microalgae cultivations were conducted under continuous illumination with photon flux densities of $200\;{\mathrm{µmol/m}}^{2}$ s for the 25 L-system and 150–$680\;{\mathrm{µmol/m}}^{2}$ s for the 200 L-PBR, using the airlift principle (12 L/min) at 22±3 °C. Light intensity of the 200 L-PBR was increased accordingly to the increasing biomass. The VIS sensor measurements were performed independent from this light source.

As cultivation medium a industrial growth medium composed of 0.5 g/L NPK fertilizer (Hauert MANNA Düngerwerke GmbH, Nürnberg, Germany; parts by weight: ${\mathrm{NaNO}}_{3}$: 3, $P_{2}O_{5}$: 15, K_2_O: 35, MgO: 5, Fe: 0.4) supplemented with additional ${\mathrm{NaNO}}_{3}$ and tap water was used. The photon flux density was increased throughout the cultivation phases. pH was kept constant at 6.8 ± 0.2 by the ${\mathrm{CO}}_{2}$-enriched air in the CellDeg system and increased from 6.5 to 7.7 ± 0.3 in the two PBR systems.

### 2.2. Analytics

The analytics were performed in parallel to the multi-wavelength VIS sensor measurements as reference data and are in accordance with, or slightly modified from, protocols used in [13].

#### 2.2.1. Biomass Dry Weight Concentration

The biomass dry weight (DW) concentration was determined by filtering 10 mL in triplicates, according to the biomass concentration of the microalgae sample, through a pre-dried and pre-weighed ($W_{1}$) glass microfiber filter with a 0.2 µm mesh size (Carl Roth GmbH KG, Karlsruhe, Germany). After filtration, filters containing biomass were washed with 20 mL deionized water; then, dried at 60 °C for 24 h in a laboratory heating cabinet (Salvis, Bender + Hobein, Zuerich, Switzerland) and weighed again ($W_{2}$). The weight of the filter containing dried biomass ($W_{2}$) and the filter without biomass ($W_{1}$) was measured using a Kern 870 balance (Kern & Sohn GmbH, Balingen, Germany). The biomass DW concentration [g/L] was calculated using Equation (1):

(1) $$c_{\mathrm{DW}}\;\left[g/L\right]=\frac{\left(W_{2}-W_{1}\right)}{10\;\mathrm{mL}}\times 1000$$

#### 2.2.2. Pigments Concentration

Chlorophyll α and β, total carotenoids, and astaxanthin were extracted using a modified method from Seely et al. [39]. The optical density of the cultures was monitored at 750 nm using a UV/VIS spectrophotometer (Thermo Fisher, Waltham, MA, USA, GENESYS 150). A sample of 1 mL of microalgal suspension was diluted with 0.9% NaCl solution to 0.7 ≤ ${\mathrm{OD}}_{750}$ ≤ 1.0 and transferred into 2 mL reaction tube and centrifuged to pellet cells. After discarding the liquid phase, the pellet was resuspended in 1 mL dimethyl sulfoxide (DMSO), and then thermoshaken for 10 min at 70 °C and 700 rpm. The tubes were centrifuged again to separate the extracts from the remaining biomass, and the supernatant collected in a fresh 2 mL reaction tube. The extraction step was repeated one more time with fresh DMSO. The absorbance of the 2 mL of DMSO extract were measured at 480, 530, 649, and 665 nm. The quantification of chlorophylls α and β and carotenoid concentrations in [mg/L] was performed using spectrophotometric equations based on Wellburn et al. [40]’s method, using the Equations (2)–(5).

(2) $$c_{Chl\;\alpha }\;\left[\mathrm{mg}/L\right]=12.19A_{665}-3.45A_{649}$$

(3) $$c_{Chl\;\beta }\;\left[\mathrm{mg}/L\right]=20.05A_{649}-5.32A_{665}$$

(4) $$c_{Chl\;total}\;\left[\mathrm{mg}/L\right]=c_{Chl\;\alpha }+c_{Chl\;\beta }$$

(5) $$c_{Car}\;\left[\mathrm{mg}/L\right]=\frac{1000A_{480}-2.14\;c_{Chl\;\alpha }-70.16\;c_{Chl\;\beta }}{220}$$

where $A_{\lambda }$ is the absorbance of the extracts at wavelength λ multiplied by the initial dilution and by the factor to account for the 1 mL of sample extracted into 2 mL of DMSO extract (Factor: 2 following the given protocol). In accordance with the methods of [41,42], the astaxanthin concentration was calculated using the absorbance at 530 nm and Equation (6).

(6) $$c_{\mathrm{Atx}}\;\left[\mathrm{mg}/L\right]=\frac{A_{530}+0.0069}{0.1783}$$

### 2.3. The Soft Sensor and Modeling Data Set Acquisition

**Figure 2:**
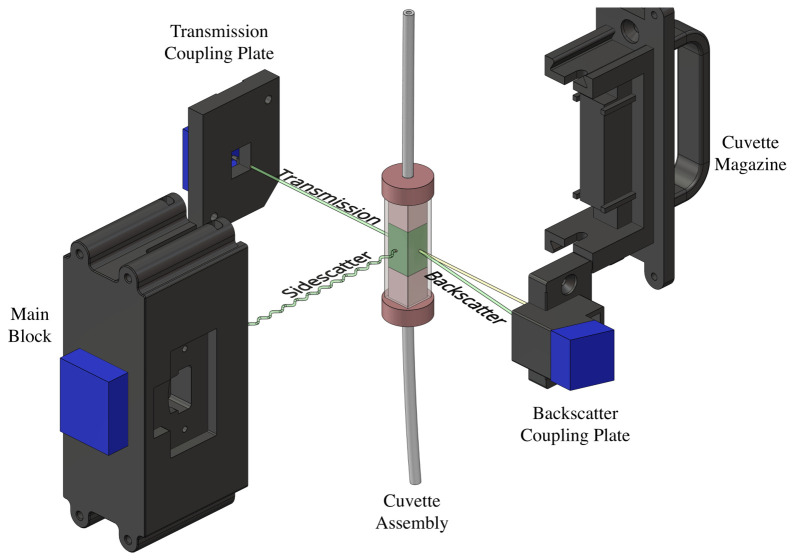
Model of the multi-wavelength VIS sensor; main block (**left**) and cuvette magazine (**right**); blue components represent photodetectors and LED array (combined with the backscatter photodetector). Geometric measurements of the main block: ≈12×8×6 cm.

The hardware sensor used in this study is based on a design developed at Leibniz University Hannover, Germany, which was further refined through additional modifications introduced in this work [9]. To provide more reliable measurements, the sensor housing (Technische Universität Dresden, Germany) was 3D-printed using black filament to minimize the reflection and scattering of the internal light with the inner structures and also to provide improved light-seal towards the outside. Additionally, the housing now consists of only two components: first, the main block, which contains all Light Emitting Diode (LED) excitation sources and photodetectors mounted in fixed positions, thereby adding stability to the LED–photodetector alignment; and second, the cuvette magazine, which holds the cuvette securely in place. The cuvette magazine is inserted laterally into the main block, allowing easy access to the cuvette and creating a light seal towards the outside, as shown in Figure 2. The hardware sensor used in this project is a multi-wavelength VIS sensor, which operates by analyzing the interaction of visible light with a biological sample running though the cuvette to gather optical information about it. The sensor, uses three LEDs serving as light sources. These LEDs emit light at distinct wavelengths: 505, 690, and 780 nm, (B3B-443-B505 for 505 nm, the LED690-03AU for 690 nm, and the LED-780-524 for 780 nm). The hardware sensor can be classified as a low-cost device, as the costs for the hardware-components required to assemble remain within a three-digit price range.

The three LEDs were selected in a way that each covers a different region of the light spectrum with a high potential for an unique optical interaction with the biomass of *C. zofingiensis*. In the range of the 505 nm LED, carotenoids exhibit their strongest absorption, with maximas between 440 nm and 490 nm, while remaining largely outside the absorption range of chlorophyll b (600–690 nm). The 690 nm LED lies within the absorption maxima of chlorophyll. The 780 nm LED, in contrast, was chosen outside the characteristic pigment absorption bands to provide an unaffected signal based solely on the particle concentration of the biomass in the sample. The multi-wavelength VIS sensor includes three photodetectors (photodiodes): one positioned directly opposite the LED-array to measure transmission, another perpendicular to the beam path to measure sidescatter, and a third placed adjacent to the LED to measure backscatter (Figure 2). The suspension flows with a distinct rate through a modified standard lab cuvette (base removed to open it at both ends), made from polystyrene. A standard lab cuvette (Kartell Labware, Noviglio, Italy; four window cuvette, 4.5 mL, 10 mm light path length; Art. 1960) was used due to its standardized dimensions and material properties to provide consistency and reliability across various experimental setups.

### 2.4. Data Collection Using the Multi-Wavelength VIS Sensor

**Figure 3:**
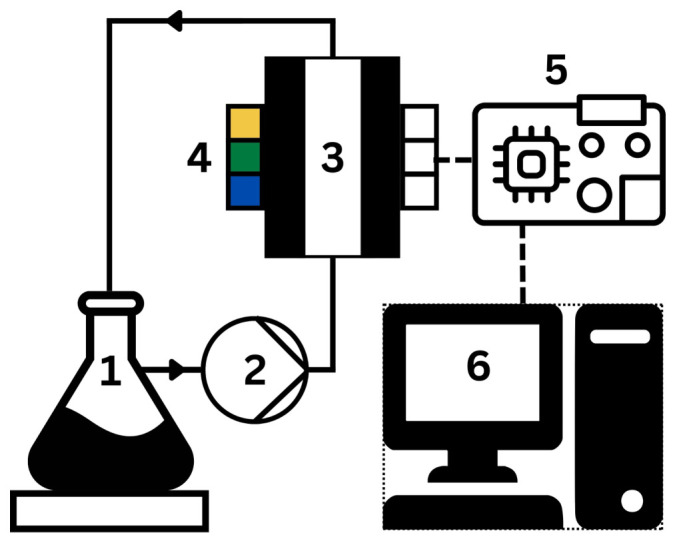
Multi-wavelength VIS sensor setup: (1) Sample placed on a stirring plate (for continuous measurement 25 L-PBR); (2) Peristaltic pump; (3) Polystyrene photometer cuvette; (4) multi-wavelength VIS sensor equipped with three LEDs and three photodetectors (5) Microcontroller setup; (6) PC to control the multi-wavelength VIS and record the data.

Although the device was designed to be integrated within an active bioreactor, continuously monitoring the microalgae cultivation, the calibration was conducted in a controlled laboratory setup. For data acquisition, 150 mL of sample was placed in a volumetric flask and agitated using a magnetic stirrer (KA Werke GmbH & Co. KG, Staufen, Germany) ensuring sample homogeneity (Figure 3(1)). The cuvette of the multi-wavelength VIS sensor was vertically connected to a peristaltic pump (Cole-Parmer™ Masterflex™ L/S, Vernon Hills, IL, USA, *7258-10*) with a pump head (Cole-Parmer™ Masterflex™ L/S, Vernon Hills, IL, USA, Easy Load II) providing a flow of 52 mL/min to the lower end of the cuvette (Figure 3(3)). Flows of 13, 26, 40, and 78 mL/min were tested as well. Each flow rate created a slight offsets within the measurement. To reduce the amount of variable parameters in the MLRM the additional flow rate were dismissed. In between different samples, the cuvette was rinsed with water and replaced when scratched or fractured to ensure clear light paths and to avoid nucleation sites for biomass accumulation and bubble adhesion.

The data collected with the multi-wavelength VIS sensor measured the optical properties of *C. zofingienis* across all permutations of the three LED wavelengths (505 nm, 690 nm, and 780 nm), the three photodetectors (backscatter, sidescatter, and transmission), and the eight current intensities for the LEDs (0.25–3.0mA). This results in a total of 72 unique combinations processed by the micro controller (Figure 3(5)). Each observation links the 72 light sensor readings to laboratory-determined values for the six response variables. The multi-wavelength VIS sensor completes a full cycle of these reading combinations in just over two minutes, during which it records five readings for each of the 72 combinations. The analysis uses the average of these five readings to provide consistency and reduce variability in the measurement. It should be noted that there were little to no fluctuations in between these five readings.

Performing four independent cultivation cycles lead to a total of 209 data points, covering a wide range of biomass and pigment concentrations. Of these, 50 datapoints originated from the 200L pilot-scale PBR, while the remaining samples were generated using 150mL laboratory-scale cultivators. The data set was split into a training data set (n = 169) to train the MLRM with and testing data set (n = 40) to validate the MLRM. A random split was used to ensure an unbiased data distribution for modeling and validation. The modeling and validation of the resulting MLRM using the testing data were performed in R (RStudio, version 4.4.1).

### 2.5. Training Data Set Preparation and Censored Data

The photodetectors recorded values ranging from 0 (no detected light) to 32,767 (maximum detectable signal). Once the measured signal approached the upper limit of 32,767 (“whiteout”), the photodetector became overexposed to light and the measurement invalid. Accounting for the noise in the data occurring near extreme readings a whiteout threshold of 32,500 was applied.

Three additional readings (one per photodetector) captured ambient light when LEDs were inactive, quantifying the blank caused by light leakage. Readings near zero (“blackout”) are contaminated with ambient light. For blackout detection, incremental offsets were systematically added to each photodetectors blank signal. The point at which the proportion of invalidated readings changed abruptly was interpreted as the transition from valid signal to noise (elbow analysis, data on request). This analysis indicated that adding an additional value of 20 to the blank readings provided a conservative and robust offset for defining the blackout threshold.

This approach ensured that only valid, noise-free data was provided for the regression modeling. The improved housing provided excellent light sealing and reduced the blank values to lower than 0.1% of the reading range of the photodetectors, extending the detection range towards lower readings of the multi-wavelength VIS sensor in the process.

**Figure 4:**
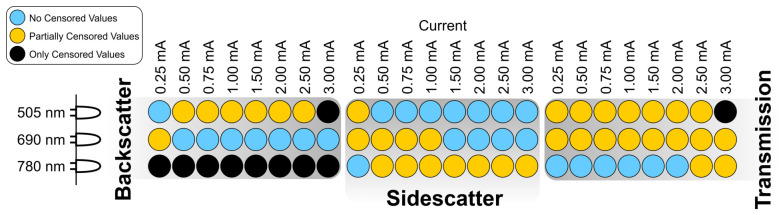
Distribution of LED-photodetetor combinations by censoring status. 72 LED-photodetector-current combinations, grouped by photodetector (backscatter, sidescatter, transmission), wavelength (505 nm, 690 nm, 780 nm) indicating censoring status: fully valid (blue), partially censored (yellow), or entirely censored (black).

Of the 72 LED-photodetector combinations, 26 yielded consistently valid readings across all samples, 36 exhibited partial censoring (blackout or whiteout), and 10 were entirely censored (Figure 4).

As expected, only a subset of the LED-photodetector combinations of the multi-wavelength VIS sensor proved suitable for modeling approaches. A valid backscatter signal from the 780 nm LED could not be detected. In contrast, valid backscatter data were obtained for the 690 nm and 505 nm LEDs. Censoring effects occurred more at very high or very low LED currents, where the emitted light either saturated the photodetector (whiteout) or fell below its detection range threshold (blackout).

As predictors for the MLRMs, the raw data from all 26 valid LED–photodetector combinations were included. In addition, transformed versions of these data were provided as supplementary predictors. Linear MLRMs preferentially incorporate predictors with linear relationships. Backscatter and transmission signals exhibited a negative exponential decay relative to all response variables. Accordingly, an “inverse-normal” transformation was applied, in which each reading was first normalized by dividing it by the training set mean of the corresponding LED–photodetector and then inverted (mean/value). The final predictor set used for modeling consisted of 26 raw data predictors and 26 inverse-normal transformed predictors, resulting in a total of 52 predictors.

## 3. Results

### 3.1. Modeling Approaches

Three modeling approaches where evaluated: Akaike Information Criterion (AIC)-based stepwise linear regression, Random Forest regression, and SVR using various kernels. These methods have been successfully applied to microbial and microalgal environmental monitoring and provide complementary insights into predictor importance [43,44,45].

AIC-based stepwise regression iteratively adds or removes predictors to improve model performance, guided by the Akaike Information Criterion. AIC-based stepwise regression balances fit quality against model complexity, penalizing excessive predictors [43].

Random Forests construct decision trees, each trained on random subsets of observations and predictors. Averaging predictions across trees reduces overfitting while capturing nonlinear relationships [44].

SVR applies kernel methods to linear regression while explicitly improving for generalization through margin-based loss functions [46]. In this study, a comprehensive hyperparameter sweep was performed, covering margin parameters ϵ∈{0.01, 0.1, 0.2, 0.5, 1} and penalty parameters C∈{0.1, 1, 10, 100, 1000} across multiple kernel types. For radial basis function (RBF) kernels, width γ∈{0.001, 0.01, 0.1, 1, 10} was evaluated, whereas polynomial kernels were tested with degrees d∈{2, 3, 4, 5} and coefficients c∈{0.0, 1.0, 2.0, 5.0}. Linear and sigmoid kernels were also included in the evaluation but consistently exhibited inferior performance.

While the objective was to construct a calibration model using the smallest set of predictors that still provided the highest accuracy, model performance was evaluated using the training data set. $R^{2}$ of the testing data set, Root Mean Squared Error (RMSE), and the testing data set Mean Absolute Error (MAE) were used as parameters for model evaluation. The MLRMs providing the best calibration performance for the soft sensor are summarized in Table 3. The mean (interquartile range) of observed responses were for Biomass DW 4.6 (1.7–6.4) g/L, Chlorophyll α 31 (13–40) mg/L, Chlorophyll β 13 (4–17) mg/L, total Chlorophyll 44 (17–60) mg/L, Carotenoids 18 (6–25) mg/L, and Astaxanthin concentration 8.7 (2–12) mg/L.

SVR-MLRMs with nonlinear kernels consistently outperformed linear approaches. For biomass DW concentration, test RMSE decreased from 0.939 (best linear model) to 0.677 (SVR polynomial). RBF and polynomial kernels achieved similarly strong performance across most responses, while linear and sigmoid kernels underperformed relative to both nonlinear SVR and AIC-based stepwise regression. For carotenoid concentration a AIC-based stepwise regression was picked based on marginal differences in the $R^{2}$ value and a lower MAE value (SVR-RBF: $R^{2}$ = 0.954, MAE = 1.911) compared to the SVR-RBF. These created MLRMs effectively calibrate the soft sensor to measure biomass and pigment concentrations of *C. zofingienis* cultures.

### 3.2. Predictor Importance Patterns

Predictor variables within each LED–photodetector-transformation class are highly correlated across different current intensities, predictor importance was assessed at the class level (i.e., LED type × wavelength × transformation status) rather than for each individual current measurements. This approach prevents artificial dilution of importance that occurs when highly correlated predictors are evaluated independently during iterative selection or permutation testing.

**Figure 5:**
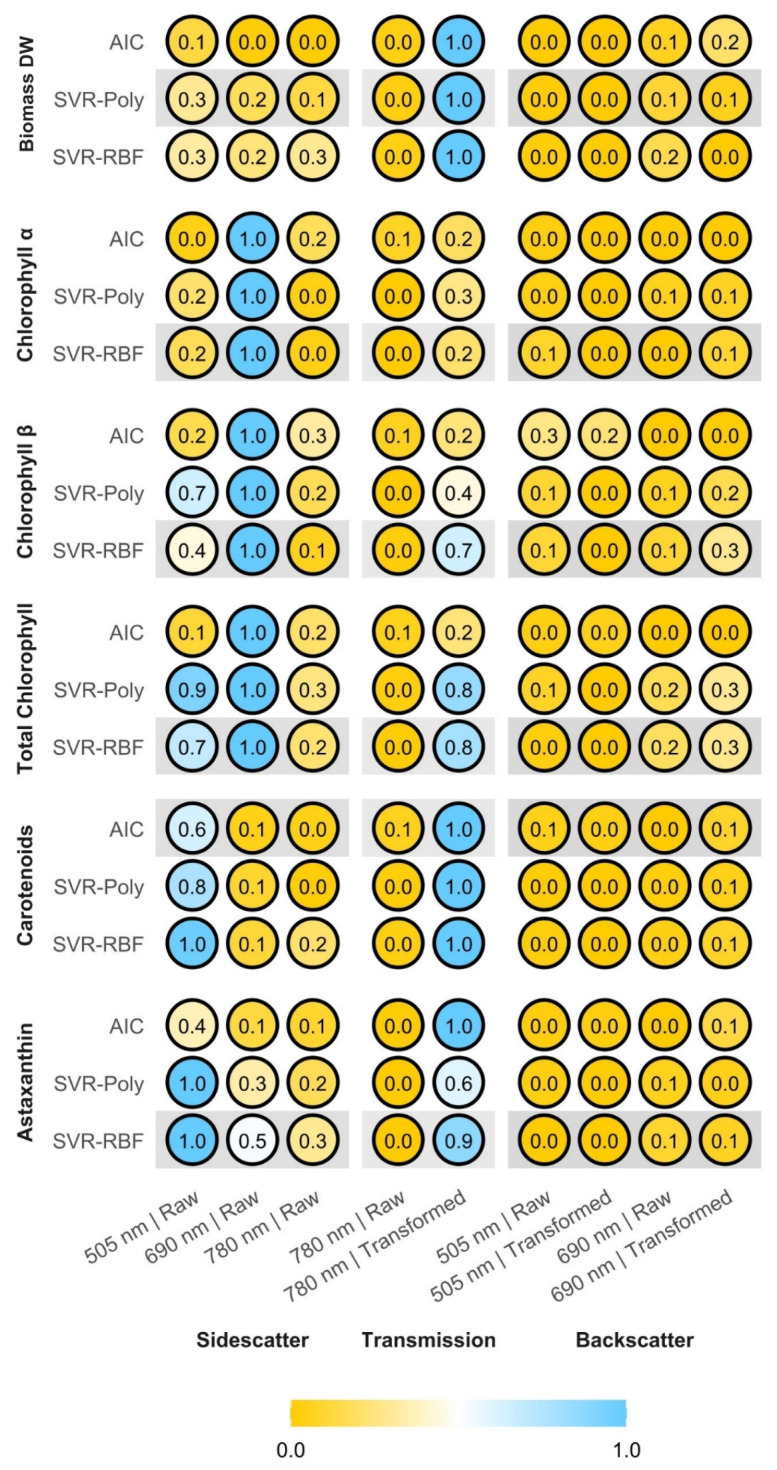
Relative importance of predictor class combinations across modeling approaches and response variables. Each facet shows normalized predictor importance (0–1 scale) for one response variable, computed via three methods: AIC stepwise (ΔAIC from class removal), SVR-polynomial (permutation ΔRMSE), and SVR-RBF (permutation ΔRMSE). Each data point represents the collective importance of all current intensities within a given soft sensor-Transformation class. Best-performing methods from Table 3 are highlighted in gray.

To quantify predictor importance across the three modeling approaches, method-appropriate techniques were applied. For the AIC-based stepwise linear regression, importance was assessed by sequentially removing entire predictor classes from the full model and measuring the resulting change in AIC (ΔAIC); larger degradation in model fit upon removal indicates greater predictor importance. For the SVR-Polynomial and SVR-RBF models, a permutation-based approach was used: all predictor values within a given class were randomly shuffled simultaneously, breaking their relationship with the response variable, and importance was quantified as the resulting increase in RMSE (ΔRMSE) relative to the unperturbed model. A larger ΔRMSE indicates that the model relied more heavily on that predictor class for accurate predictions. Importance values were then normalized to a 0–1 scale within each method-response combination to enable comparison both across predictors within a single model and across the three modeling approaches. Figure 5 pictures the relative predictive contribution of each predictor to the three MLRMs approaches for comparison of which optical configurations provides the most informative signal for each MLRM.

Predictor importance patterns were consistent across modeling methods for most responses (Figure 5). For biomass DW concentration, all three methods unambiguously identified transmission 780 nm (transformed) as the sole critical predictor (importance = 1.0), with all other predictors contributing negligibly. This finding aligns with standard turbidity measurement practices, where transmission provides a direct proxy for cell density that follows, e.g., Beer–Lambert behavior after inverse normal transformation. The fit of the achieved model, with $R^{2}$ = 0.975, is comparable to calibrations obtained with other transmission-based VIS sensors, such as the 980 nm transmission cuvette used by [30] or the multi-wavelength transmission flow cell described by [34], achieving $R^{2}$ values of 0.995 and up to 0.99. The data-driven approach presented by [18] achieved a $R^{2}$ of 0.8267 correlating a predictors of a multi-wavelength camera sensor with the biomass DW concentration. For Chlorophyll α, all methods converged on sidescatter 690 nm (raw) as the dominant predictor. Chlorophyll β showed similar dependence on sidescatter 690 nm (raw), though the best-performing method (SVR-RBF) also benefited from transmission 780 nm (transformed, importance = 0.7). Total chlorophyll naturally reflected the combined importance patterns of its α and β components. The strong performance of 690 nm sidescatter for chlorophyll prediction corresponds to chlorophyll absorbance maxima in the red spectral region (chlorophyll α at 665 nm, chlorophyll β at 645 nm).

Prediction of the carotenoid concentration relied primarily on transmission at 780 nm (transformed) across all methods, with secondary contributions from sidescatter 505 nm (raw, importance ≥ 0.6). Astaxanthin showed the most complex importance pattern: the best-performing model (SVR-RBF) weighted sidescatter 505 nm (raw, importance = 1.0) and transmission 780 nm (transformed, importance = 0.9) nearly equally. Total carotenoid and astaxanthin concentration are biomass DW associated variables, accordingly 780 nm (transformed) associated with the biomass DW concentration contributes to these MLRM strongly as well. The critical role of the 505 nm measurement for the prediction of the carotenoid and astaxanthin concentration reflects absorbance peaks in the 430–480 nm spectral range characteristic of carotenoid chromophores, with astaxanthin exhibiting particularly strong absorbance near 480 nm [47]. The high importance of the 505 nm (raw) sidescatter predictor for carotenoid and astaxanthin concentration predicting MLRMs provides the prove for the importance of the selection of suitable emission diodes for the soft sensor.

Backscatter photodetectors contributed only marginally to the MLRMs across all variables. This suggests that, given the available transmission and sidescatter data, predictive MLRM can be constructed without the backscatter signal. In addition, for the 780 nm LED, all values across all current levels resulted in whiteout signals (Figure 4). Possible reasons for the persistent overexposure include an overly sensitive photodetector, direct reflection of the 780 nm LED emission at the cuvette glass into the photodetector, or insufficient optical separation between the illumination path of the 780 nm LED and the backscatter photodetector. Any of these factors could result in constant saturation of the LED–photodetector combination and cause whiteout of all measurements. Consequently, light paths and housing design should be assessed and a less sensitive photodetector considered for future multi-wavelength VIS sensor designs.

### 3.3. Performance of the Soft Sensor Operating Continuously in the Model Process

To test the capabilities and limitations of the soft sensor under realistic operating conditions and to acquire data independent from the used training dataset and testing dataset, an additional 7-day cultivation was carried out in the 25 L-PBR system equipped with the soft sensor. The cultivation was also performed in a different cultivation system used to generate the training data set to test for overfitting. Offline measurements were performed in parallel to evaluate the performance of the MLRMs. The soft sensor operated continuously with periodic measurements every 30 min. During the cultivation, the cuvette was replaced after one, two, and three days of the cultivation. At days five and six, the cuvette was back-flushed. A back-flushing cycle consists of reversing the flow direction through the cuvette for 5 min. The accumulation of biomass and adhesion of bubbles was successfully prevented by rinsing the cuvette with water and replacing it in time to ensure a clear light paths [48].

**Figure 6:**
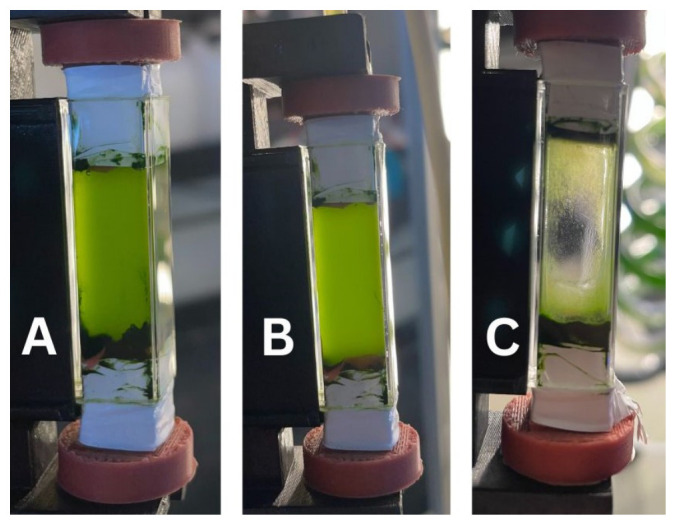
Biomass accumulation in the sensor cuvette (**A**): cuvette in continuous operation for two days; biomass concentration ∼1.2 g/L. (**B**): cuvette after 5 min of back-flushing following two days; biomass concentration ∼1.2 g/L. (**C**): cuvette after four days and two back-flushing cycles.

Examples for the accumulation of biomass in the cuvette are shown in Figure 6. The amount of biomass accumulated depended on the biomass DW concentration of the culture. As illustrated in Figure 6A, after two days of continuous operation the light path in the center of the cuvette remained completely clear, although a substantial amount of biomass had accumulated at the lower end of the cuvette. After 5 min of back-flushing, most of the deposited biomass was removed (Figure 6B), allowing the cuvette to operate for an additional two days with occasional back-flushing. After four days, a biofilm began to form towards the middle part of the cuvette into the light path of the sensor (Figure 6C), making replacement of the cuvette necessary. In the given setup, the sensor should be inspected daily for potential biofilm formation. Early detection of biofilm development could be integrated into the existing optical configuration as an additional response variable, optional in combination with a dedicated measurement module incorporated into the sensor assembly. Regular automatic back-flushing (after each measurement cycle or after a predefined number of operating hours) could substantially reduce biofilm formation, lower handling effort, and decrease the frequency of cuvette replacement.

**Figure 7:**
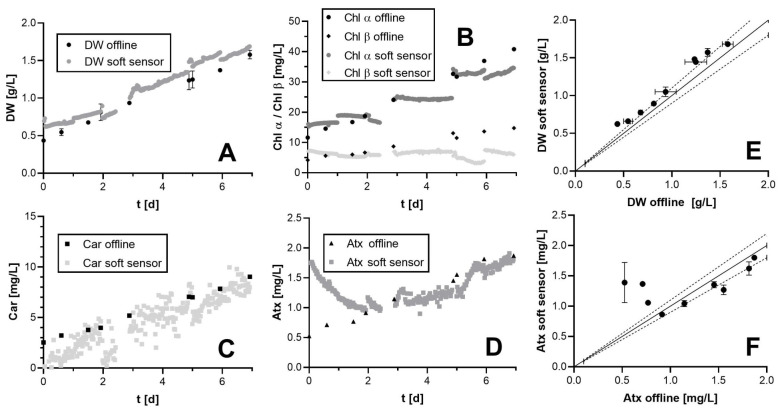
Validation of continuously operating soft sensor with offline measurements during a 7-day cultivation of *C. zofingiensis* in a 25 L-PBR system. (**A**): Comparison between biomass DW concentration offline measurements and model predictions based on continuous operation. (**B**): Comparison between chlorophyll α and β (Chl α, Chl β) concentration offline measurements and model predictions based on continuous operation. (**C**): Comparison between carotenoids (Car) concentration offline measurements and model predictions based on continuous operation. (**D**): Comparison between astaxanthin (Atx) concentration offline measurements and model predictions based on continuous operation. (**E**): Parity plot comparing biomass DW concentration offline measurements (standard diviation, n = 3) with model predictions under continuous operation. (**F**): Parity plot comparing astaxanthin (Atx) concentration offline measurements ( standard diviation, n = 3) with model predictions under continuous operation. Parity plot include the line of equality and a 10% tolerance band.

The results of all measurements obtained offline in comparison to the soft sensor measurements are shown in Figure 7. The measurements of the soft sensor are the predictions of the best performing MLRM (Table 3).

The soft sensor measurements were able to quantify the biomass DW concentration with high precision (Figure 7A). The Standard Error of the Mean (SEM) was calculated as ±0.079 g/L, which places most soft sensor measurements within the 10% tolerance band, as shown in Figure 7E. These results are comparable to the empirical calibrations reported by Marxen et al. [28] and Sandnes et al. [29], who achieved accuracies of 8% and 10%, respectively, using a transmission-based soft sensor at similar biomass DW concentrations ranging from 0.5 to 2.2 g/L. In Figure 7A, the impact of cuvette cleanings and replacements is visible as stepwise shifts in the sensor measurement. However, the biomass DW concentration measurements obtained by the soft sensor do not appear to be affected by biomass accumulation inside the cuvette. Even over an extended interval without cleaning (day three to day five), the soft sensor signal showed no noticeable deviations from the trend of accumulating biomass. This suggests that the observed steps in the graph are primarily caused by the handling of the cuvette rather than by biomass accumulation in the cuvette. Although the cuvette fits tightly into the sensor housing and should ideally maintain a constant position, small positional deviations seem to occur when the cuvette is accessed for back-flushing or replacement and can impact the multi-wavelength VIS sensors biomass DW concentration measurements.

In contrast the measurement of chlorophyll concentration appears to be strongly affected by biomass accumulation in the cuvette, as shown in Figure 7B. This may be caused by the reliance of the MLRMs on the 690 nm sidescatter measurement. The measurements of the continuously operating sensor do not reflect the steadily increasing chlorophyll concentration, particularly at higher levels. After cuvette replacement or a back-flushing cycle, distinct steps of increment are clearly visible for both chlorophyll α and chlorophyll β. Measurements of the total chlorophyll concentration exhibit similar behavior. Overall, the quantification of the chlorophyll concentration under these conditions must be considered unreliable, especially at higher biomass concentrations.

The measurement of the carotenoid concentration was not affected by cuvette cleaning procedures as the chlorophyll concentration measurements were. However, the variance of the data is substantially higher compared to the other quantified parameters, and at lower concentrations a clear underestimation of the carotenoid concentration is evident (Figure 7C). The increased variance in the measurement data (MLRM-predictions) may be attributable to the modeling approach selected for the prediction of the carotenoid concentration (AIC-based stepwise). In contrast to the SVR-based models, small variation in sensor input results in a corresponding change in the predicted carotenoid concentration. In addition to considering an alternative modeling strategy, smoothing of the soft sensor signal combined with shorter measurement intervals may further enhance accuracy. The slight underestimation observed at low concentrations may result from measurements conducted outside the trained modeling range (>6 mg/L). Expanding the model by incorporating additional training data within this concentration range is expected to improve predictive accuracy and overall robustness.

As shown in Figure 7D, the soft sensor measurements of the astaxanthin concentration are representative only within a limited concentration range. At lower concentrations, the model substantially overestimates the astaxanthin concentration. In particular, the SVR-RBF model is unable to provide accurate predictions outside its trained concentration range (Table 3), primarily due to the presence of extreme points in this region. The model may be readily improved by incorporating additional training data, particularly in the lower concentration range. At higher concentrations (>1 mg/L), the soft sensor estimates the astaxanthin concentration reliably, with most values falling within the 10% tolerance band (Figure 7F). The corresponding SEM of 0.0562 mg/L further indicates a stable and reproducible measurement performance in this range. The smooth prediction trends produced by the SVR-based MLRMs reflect an inherent characteristic of SVR: by optimizing within an epsilon-insensitive loss function, SVR treats small deviations from the regression surface as acceptable and does not fit them, effectively separating the underlying biological signal from measurement noise [45]. Linear regression MLRMs, by contrast, fit directly to all observed variance in the training data, meaning natural measurement noise is incorporated into the model structure and propagates through to the predictions. Both behaviors are characteristic of their respective methods and represent a fundamental trade-off between noise, tolerance, and strict data fidelity.

## 4. Conclusions

From the viewpoint of a user, the sensor measurements provide a reliable and continuous indication of biomass-related process dynamics. The real-time nature of the measurements enables timely operational decisions, particularly for trend-based adjustments and early anomaly detection. Most notably were the robust predictions of DW and astaxanthin concentrations measured under continuous conditions, with those estimates falling within a suitable range for practical bioprocess applications. The validation with independent data from a cultivation system not used for model development strongly indicates that the models are not overfitted. Compared to conventional turbidity probes or NIR-based biomass sensors, the system offers higher flexibility due to the machine-learning-based calibration estimating different characteristics of the biomass like pigmentation in real-time. The use of three photodetectors and LEDs further increases robustness compared to single-wavelength, single-detector configurations. If one measurement becomes unreliable, for example transmission at high biomass concentrations, the remaining detectors still provide stable measurement information. Nonetheless, classical turbidity probes may outperform it in robustness for the measurement of low biomass concentrations.

Limitations of the soft sensor remain within the measurement accuracy and range. They are expected to be enhanced by providing more training data, especially for high biomass and pigment concentrations. Occasional periodic re-calibration or offline reference measurements may be required to verify the long-term validity of the sensor readings. Irregular bubbles or bigger particles with the suspension and biofilm formation within the sensor remain critical operational issues.

The reliability may be enhanced by improving cuvette fluid dynamics or automated back flushing cycles to reduce biomass accumulation and biofilm formation. For the current system, a reduction to a configuration measuring the transmission at 780 nm combined with sidescatter at 690 nm and 505 nm would retain the essential predictive information required for all six response variables. This sensor setup should be suitable for all green microalgae containing mainly chlorophylls and carotenoids.

The choice of the modeling approach substantially influences system complexity. Linear methods (AIC-based stepwise) require data transformation but can achieve competitive performance when appropriate pre-processing is applied. Nonlinear approaches such as SVR operate effectively on raw sensor outputs, potentially reducing hardware demands when transformations are difficult to implement. Nevertheless, incorporating transformed predictors like for transmission at 780 nm, improved the performance of the best MLRMs for several response variables.

The study shows that beyond established biomass measurements, data-driven sensors offer the capability to quantify additional types of biological parameters like pigment concentration in real-time. The developed sensor platform is highly versatile and can be equipped with different LEDs or photodetectors depending on experimental requirements to detect other types of natural pigments or components of the biomass. Moreover, the system enables rapid acquisition of a large amount of culture data and supports modeling approaches that provide real-time biological insights, offering the potential for integration into model-based or predictive control strategies for biological processes.

## Figures and Tables

**Table 1:** Optical monitoring methods for microalgae biomass concentration with the potential for on-line and in-line measurements.

Measured Parameter	Monitored System	Sensor Type	Calibration Approach	Ref.
Transmission/Scattering	*Scenedesmus quadricauda*	build in cuvette, 680 nm and 735 nm	empiric multi-factorial	[25]
Transmission/Scattering	*Cyanothece* sp.	build in cuvette, 680 nm and 735 nm	empiric multi-factorial	[26]
Transmission	*Nannochloropsis oculata*	turbidity probe, 840–910 nm	empiric	[27]
Transmission	*Synechocystis* sp.	turbidity probe 870 nm	empiric	[28]
Transmission	*Nannochloropsis oceanica*	flow cell, 880 nm	empiric	[29]
ISM	*Chlamydomonas reinhardtii*	flow-through microscope probe	morphology-based cluster correction	[21]
Color analysis	*Chlorella homosphaera*	RGB sensor	empiric	[17]
Transmission	*Tetraselmis viridis, Limnospira platensis*	flow cell, 940 nm	empiric	[30]
Scattering	*Chlamydomonas reinhardtii*	Focused beam reflectance probe	neural network	[31]
Raman Spectrometer	*Parachlorella kessleri*	Raman flow cell	statistical model based correlation	[32]
Scattering	*Chloromonas typhlos, Porphyridium purpureum*	Nephelometric flow cell	empiric	[33]
Color analysis/luminance	various green algae	build in dual camera	Data- driven modeling	[18]
Transmission	*Scenedesmus obliquus*	flow cell, 400, 680, 750, 850 and 940 nm	empiric	[34]

**Table 2:** Optical monitoring methods for pigment concentrations in microalgae cultivations with the potential for on-line and in-line measurements.

Measured Parameter	Monitored System	Sensor Type	Calibration Approach	Ref.
Florescence	Chlorophyll florescence of *Scenedesmus quadricauda*	build in cuvette exitation: 455 nm and 625 nm	empiric	[25]
Transmission/Scattering	Chlorophyll concentration of *Cyanothece* sp.	build in cuvette 680 nm and 735 nm	empiric multi-factorial	[26]
Raman Spectrometer	Carotenids of *Parachlorella kessleri*	Raman flow cell	statistical model based correlation	[32]

**Table 3:** Performance of the optimal MLRMs for each response variable. SVR with radial basis function (RBF) or polynomial kernels. Hyperparameters: *d* = polynomial degree, *c* = coefficient, *C* = penalty, ϵ = epsilon (margin), γ = gamma (RBF width). Training $R^{2}$ computed by training data set of 169 samples (k = 5); RMSE and MAE computed using the testing data set of 40 samples.

Response	Best Method	Hyperparameters	Training $R^{2}$	Test RMSE	Test MAE
Biomass DW	SVR-Poly	d=4, c=1, C=0.1, ϵ=0.1	0.975	0.677	0.465
Chl α	SVR-RBF	γ=0.1, C=10, ϵ=0.01	0.944	9.776	5.413
Chl β	SVR-RBF	γ=0.1, C=100, ϵ=0.01	0.864	5.919	3.303
Chl total	SVR-RBF	γ=0.01, C=1000, ϵ=0.01	0.958	7.276	5.521
Carotenoids	AIC	N/A	0.967	2.543	1.840
Astaxanthin	SVR-RBF	γ=0.01, C=100, ϵ=0.01	0.981	1.106	0.797

## Data Availability

Data will be made available on request.

## Abbreviations

The following abbreviations are used in this manuscript:

AIC	Akaike Information Criterion
DW	Dry weight
IR	Infrared
LED	Light emitting diodes
MAE	Mean Absolute Error
MLRM	Machine-learning regression model
PBR	Photo bio reactor
RBF	Radial basis function
RMSE	Root Mean Squared Error
SVR	Support Vector Regression
UV	Ultraviolet light
VIS	Visible light
